# The right thalamic ventral posterolateral nucleus seems to be determinant for macrosomatognosia: a case report

**DOI:** 10.1186/s12883-020-01970-3

**Published:** 2020-10-28

**Authors:** Amir H. ElTarhouni, Laura Beer, Michael Mouthon, Britt Erni, Jerome Aellen, Jean-Marie Annoni, Ettore Accolla, Sebastian Dieguez, Joelle N. Chabwine

**Affiliations:** 1 Division of Neurorehabilitation, Fribourg Hospital, Meyriez, Switzerland; 2 Neurology Unit, Department of Neuroscience and Movement Science, Medicine Section, Faculty of Science and Medicine, University of Fribourg, Chemin du Musée, 5, CH-1700 Fribourg, Switzerland; 3 Department of Radiology, Fribourg Hospital, Riaz, Switzerland

**Keywords:** Macrosomatognosia, Alice in wonderland syndrome, Thalamus, Stroke, Ventral posterolateral nucleus, Diffusion tractography

## Abstract

**Background:**

Macrosomatognosiais the illusory sensation of a substantially enlarged body part. This disorder of the body schema, also called “Alice in wonderland syndrome” is still poorly understood and requires careful documentation and analysis of cases. The patient presented here is unique owing to his unusual macrosomatognosia phenomenology, but also given the unreported localization of his most significant lesion in the right thalamus that allowed consistent anatomo-clinical analysis.

**Case presentation:**

This 45-years old man presented mainly with long-lasting and quasi-delusional macrosomatognosia associated to sensory deficits, both involving the left upper-body, in the context of a right thalamic ischemic lesion most presumably located in the ventral posterolateral nucleus. Fine-grained probabilistic and deterministic tractography revealed the most eloquent targets of the lesion projections to be the ipsilateral precuneus, superior parietal lobule,but also the right primary somatosensory cortex and, to a lesser extent, the right primary motor cortex. Under stationary neurorehabilitation, the patient slowly improved his symptoms and could be discharged back home and, later on, partially return to work.

**Conclusion:**

We discuss deficient neural processing and integration of sensory inputs within the right ventral posterolateral nucleus lesion as possible mechanisms underlying macrosomatognosia in light of observed anatomo-clinical correlations. On the other hand, difficulty to classify this unique constellation of Alice in wonderland syndrome calls for an alternative taxonomy of cognitive and psychic aspects of illusory body-size perceptions.

**Supplementary information:**

**Supplementary information** accompanies this paper at 10.1186/s12883-020-01970-3.

## Background

Body schema disorders (BSD) constitute a broad group of neuropsychological dysfunctions altering perceptions or beliefs regarding the bodily self [1]. They include disownership for specific body parts (somatoparaphrenia), feelings of duplicated limbs (supernumerary phantom limb), increased/decreased size of body parts (macro−/microsomatognosia) and illusory self-perceptions regarding the whole body (autoscopic phenomena). Focal brain lesions associated with such disorders can be located in an extended network predominantly involving the right hemisphere, including parietal, frontal and temporal areas. Patients presenting with BSD are often quite unique in their clinical presentation, hence the need for careful documentation of each case and neuroanatomical correlates.

Macrosomatognosia (MSG) is the proprioceptive, tactile and sometimes visual illusory experience that part of one’s body has substantially grown in size [2], also classified as “Alice in wonderland syndrome” (AIWS) in the literature [3, 4]. Illusory body size distortions have been historically related to vestibular disorders and referred to as “hyperschematia” [5]. They are widespread in literary accounts and can be studied experimentally in healthy subjects [6]. In neurological patients, the sensation of body parts enlargement can be quite striking and disturbing, but is still usually recognized as illusory [7].

Here we present a new case of left-sided MSG with unusual features: a quasi-delusional conviction that the left arm’s circumference really increased (despite objective evidence to the contrary), a chronic duration of MSG despite regression of most other associated symptoms, and a non-cortical-causing lesion (ischemic stroke in the right thalamic ventral posterolateral (VPL) nucleus). We also offer, for the first time, fine-grained tractographic evidence for a thalamo-cortical mechanism in MSG. Because this case could not be properly classified, we propose AIWS as a spectrum of symptoms spanning from one cognitive (somesthetic) extreme to a more psychic (delusional) one.

The present study was conducted in compliance of all local and international ethical rules, after the patient gave his informed and written consent.

## Case presentation

### First symptoms and management

**Fig. 1:**
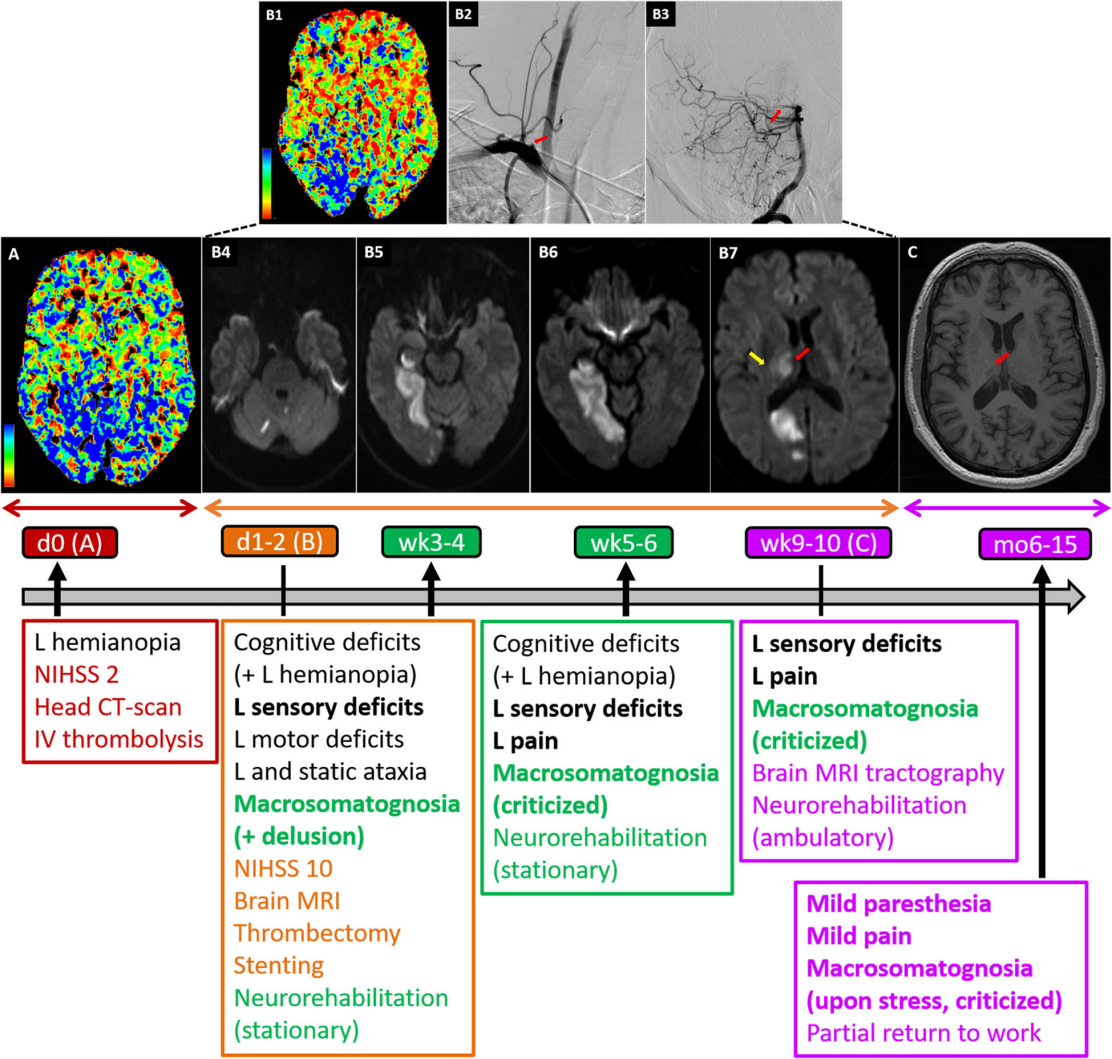
Standard brain imaging and symptom timeline. **a** *Perfusion head CT-scan performed on arrival in the emergency department (day 0)*. The Time-To-Maximum (TMax, color code at bottom left) was prolonged predominantly in the right internal occipital region and the right thalamus while the cerebral blood volume remained normal overall (not shown), thereby defining a penumbra. **b** *Brain images performed upon neurological aggravation (day 1 and day 2)*. Perfusion head CT-scan (B1) (day 1) showing increased TMax (i.e. penumbra) in the right posterior cerebral artery (PCA) vascular territory (color code at bottom left) due to new occlusion of the right PCA. A conventional arteriography performed at day 2 in order to repermealize the previously occluded right vertebral (B2, anterior view of the right subclavian artery angiography with ostial occlusion of the right vertebral artery, red arrow) by thrombectomy, allowed visualization of the PCA occlusion (B3, lateral view of the right vertebral artery and the basilar artery with the occluded P1 segment of the right posterior cerebral artery, red arrow). The Diffusion Weight Imaging sequence of brain Magnetic Resonance Imaging done subsequently the same day showed several restriction areas in the cerebellum (B4), right hippocampal and parahippocampal regions (B5), right lingual gyrus (B6), right occipital lobe (B7), right thalamus (B7, red arrow) and minor involvement of the right internal capsule (B7, yellow arrow), confirming acute ischemic lesions. **(C)** *3 T Brain Magnetic Resonance Imaging (10 weeks after stroke)*. On the T1 sequence (the section shown is approximately on the same level as on the B7 panel), the occipital lesion is not visible anymore, contrary to the right thalamic residual lesion (red arrow). *Bottom panel*: *The timeline of the main clinical finding evolution* (until the end of follow up, grey horizontal arrow) in parallel with respective brain imaging (letters in () next to time points correspond to figures in upper panels) and management strategies. Color codes relate to different treatment places, in relation with respective time points (ED at day 0 in dark red; tertiary hospital at day 1–2 post-stroke in orange; ambulatory follow up from week 9–10 until the 15th month, in purple), symptoms (symptoms of interest are in bold) and brain imaging (left-right arrows below brain imaging panels relate to the same timeline as in the text boxes below), while vertical black arrows correspond to change of management place (admission, transfer or discharge). Initial symptoms that persisted through the follow up are written in black. Detailed description of macrosomatognosia and sensory deficits was done during stationary neurorehabilitation. Macrosomatognosia and sensory deficits significantly decreased 6 months after stroke

This 45-year old right-handed man was admitted in the emergency department (ED) for sudden left visual field disturbances and left-sided paresthesia associated with right occipital headache that occurred ~ 7 h ago, after a 2–3-min transient episode of general discomfort including vertigo, black veil and left facial tingling. The patient’s medical history was otherwise remarkable for smoking, obesity, arterial hypertension, dyslipidemia, gout and childhood psychological trauma from which he totally recovered several years ago under long-term psychotherapy. Neurological evaluation on arrival (day 0) showed only moderate left homonymous lateral hemianopia (NIHSS 2). Head CT-scan (General Electrics® scanner) performed with the stroke protocol on arrival (day 0) noticed no ischemic core lesion, but a right temporo-occipital penumbra (Fig. 1a) and a 4.5 cm-long occlusion of the right vertebral artery in the V1 portion (still visible at day 2, see Fig. 1, panel B2). The patient benefited from intravenous thrombolysis with alteplase.

About 12 h after occurrence of the left hemianopia and accompanying symptoms (day 1), the patient deteriorated with new left-sided sensory deficits, left hemiparesis and cerebellar ataxia in addition to left hemianopia (NIHSS 10) and was, for this reason, referred to the closest tertiary hospital. A new perfusion Head CT-scan showed penumbra in the right posterior cerebral artery (PCA) territory (Fig. 1, panel B1), due to occlusion of the PCA (Fig. 1, panel B2). Brain MRI (Siemens®, 3 Tesla scanner) including Diffusion Weighted Imaging (DWI) sequences (5 mm slice thickness) showed restrictions in the right lateral thalamus, the right hippocampus, the right lingual and parahippocampal gyri, and the right cerebellum (not shown). About 24 h after neurological worsening (day 2), arterial thrombectomy and stenting of the proximal portion of the right vertebral artery were performed. A new brain MRI obtained during thrombectomy showed new diffusion restrictions compatible with acute ischemic insults in right occipital regions (with discrete haemorrhagic transformation), punctiform ischemia in left and right cerebellar hemispheres, in addition to the previously noticed lesions (Fig. 1, panels B4–7). A minor involvement of the right internal capsule was also suspected (Fig. 1, panel B7). Although thrombectomy was successful, leaving only a residual distal occlusion of the right PCA, no clinical improvement was noticed.

When discharged back to the peripheral hospital (day 5), the first cognitive assessment showed mild executive dysfunction, non-lateralized slowing down of visual stimuli processing, left hemianopia without evidence for hemineglect, and isolated difficult lexical access for proper names (no language deficit was noticed). The MoCA test score was 26/30. Stroke workup revealed sleep apnea, increased LDL (3.86 mmol/l), a patent foramen ovale that was finally analyzed as non-contributive to the occurrence of stroke. There was no diabetes, no systemic inflammatory disease, infection, tumor or thrombophilia. Cardiac rhythmic monitoring showed no significant abnormality and the cardiac function was normal. Thus, the etiology of stroke remained undetermined. A double anti-platelet treatment (Aspirin 100 mg/d and Clopidogrel 75 mg/d) was initiated for 3 months (due to stenting) and then a monotherapy (Aspirin) was planned for long term. The patient started Atorvastatin 40 mg/d, stopped smoking, and had continuous positive airway pressure (CPAP) therapy for sleep apnea.

### Symptom description (including MSG) and clinical findings in Neurorehabilitation

On admission in Neurorehabilitation division (20 days post-stroke), the patient had left arm heaviness with paresthesia corresponding, on neurological examination, to left-sided hypoesthesia in all modalities (touch, pain, temperature), hypo-pallesthesia, severely disturbed sense of position and mild left upper limb hemiparesis and cerebellar ataxia were still present. Neuropsychological deficits remained unchanged, but did not interfere daily activities.

Besides, the patient complained about a strange feeling of increased size (i.e. MSG) mainly in his left upper limb and left flank (see the illustration in the supplementary “figure macrosomatognosia” panel A). Although no difference in arm size was evidenced on examination and notified to him, the patient persistently declared that his feeling of increased limb size was real, showing for example, as a proof, that his shirt leaves were tighter on the left compared to the right side (no objective difference was observed by the medical team). He further described his sensation, saying: “there is a space on the left side and it feels precisely like a balloon beneath my arm when I press on it. … If I drop my arms at rest, here [showing the left arm] I have the impression that my arm hangs like this [he laterally raises his right arm up to horizonal position about 90 °], as if I am walking with a friend in the street arm in arm; it feels the same [because] the entire left side is inflated like a balloon [he shows with the right hand, the entire area along the lateral left trunk starting from the armpit]” . Closing eyes or looking in the mirror did not modify MSG, while touching or moving the affected limb (see the supplementary “figure macrosomatognosia” panel A) enhanced it. The patient did not report (even upon proactive questioning) any feeling of illusory, supernumerary or disowned limb, and did not show particular emotion towards inflated body parts. There was no sensation of splitting of the self either. Rather, he reported mislocation of sensory stimulation during neurological assessments (e.g. he perceived a light left forearm tactile stimulation on the left back side of his neck).

One to 2 weeks upon admission in stationary neurorehabilitation (i.e. 4–5 weeks post-stroke), he could still precisely describe MSG in the neck, around the left eye, the left ear and the whole left side of the neck, the left arm and trunk (see the supplementary “figure macrosomatognosia” panel B). Otherwise he specified experiencing it in particular when he touched or mobilized those areas. This last statement has to be taken with caution as the patient still reported spontaneous MSG in his left arm and left flank. He possibly meant that MSG in additional areas did not exist at rest). Sensory disorders similarly worsened with movement. As the examiner started passively moving the patient’s left upper limb from a 90° external abduction towards the left flank, the patient felt the arm heavier (he had his eyes closed during this evaluation). He started feeling that his left arm pushed against the trunk “balloon” at ~ 80° abduction and this sensation increased with greater adduction, becoming more and more unpleasant until the task had to be stopped around 45° abduction. When the patient opened back his eyes, his limb position did not correspond to what he expected when his eyes were closed. A formal psychiatric evaluation ruled out any active psychiatric symptom or disease.

### Brain MRI with diffusion Tractography

The last brain MRI, done ~ 10 weeks post-stroke (General Electrics® Discovery MR750, 3 Tesla scanner) with DWI sequence for tractography purposes, displayed only a right thalamic lesion that had decreased in size and coincided with the VPL (T1 sequence, 1 mm slice thickness; Fig. 1c). Detailed data acquisition and analysis procedures are found in supplementary material (see Diffusion tractography methods). In brief, a lesion mask centered on the thalamic lesion was manually drawn from the T1 and T2 volumes, then used as a seed for ensuing tractography. For visualisation purposes and cross validation, the probabilistic analysis was completed by a deterministic reconstruction of fiber tracts constrained to a few target areas associated to MSG, and/or anatomically or functionally connected to the VPL according to previous reports [8–12].

**Fig. 2:**
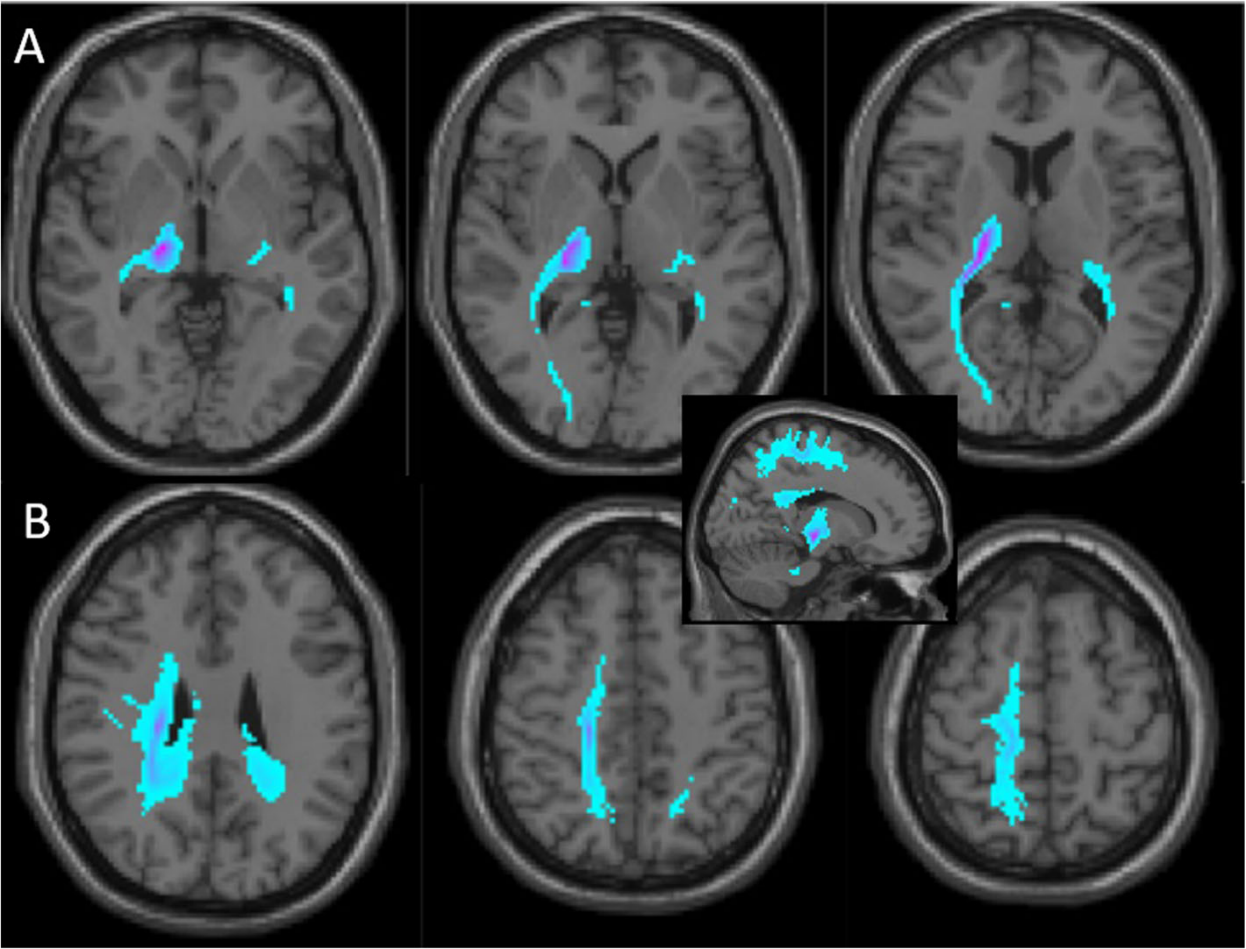
Probabilistic connectivity of the right thalamic lesion based on Magnetic Resonance Imaging (Diffusion Tensor Imaging). This analysis is overlayed on the same T1 brain image as in the Fig. 1c. Blue clusters disclose areas to which the lesion was expected to be connected (darker color indicates stronger connectivity), independently from the actual number of residual fibers. **a** Successive sections from the lesion bottomward show connectivity with the ipsilateral occipital region. **b** In more superficial sections, lesion connectivity points to a superficial linear paramedial area spanning from the ipsilateral frontal cortex through the superior parietal lobule, including also the primary somatosensory cortex (middle and most right panels). More deeply and internally, the inferior and anterior precuneus regions seem to be involved (most left panel). The **inset** corresponds to a mesial view further confirming the antero-posterior extension of the most superficial (frontal, parietal cortices and superior parietal lobule) and the deepest (precuneus) connections of the thalamic lesion

**Fig. 3:**
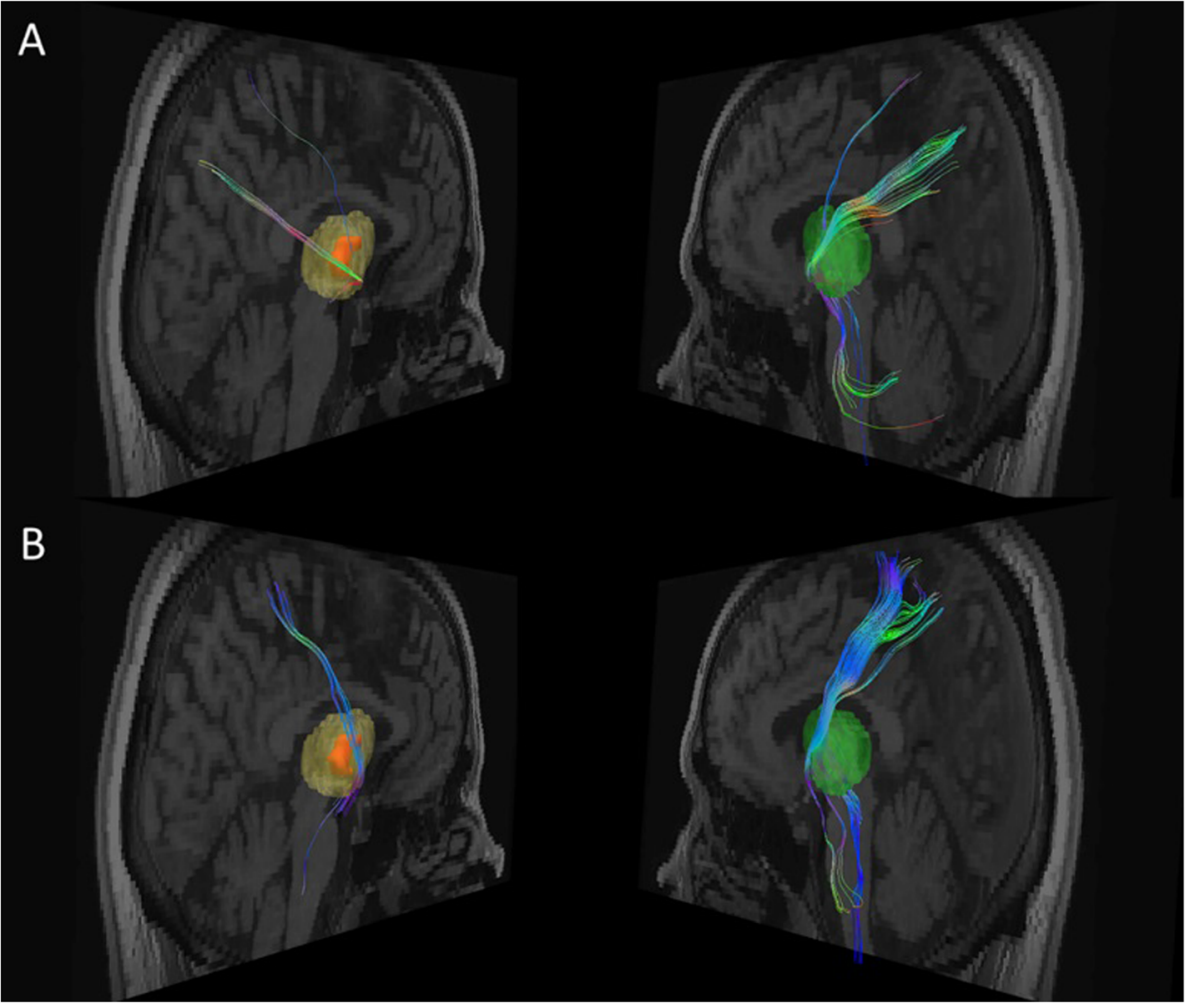
Fiber tracts connection between the right thalamic lesion area and respectively the precuneus (**a**) and the superior parietal lobule (**b**) from Brain Magnetic Resonance Imaging (Diffusion Tensor Imaging). Data were extracted from the same T1 brain image as in the Fig. 1c. The left panels show respectively a 3D reconstruction of the right thalamic lesion (orange) superimposed over the whole thalamic mask (in light yellow), and residual fibers. Their symmetric counterparts are shown in the right panels with the mask of the whole left thalamus (light green) and fiber tracts. White matter fibers colors code corresponds to their directions. Reduction in the number of fiber tracts through the lesion is visible for both targets of interest: 80% for the precuneus (**a**) and 90% for the superior parietal lobule (**b**)

A whole-brain unconstrained probabilistic tractography (Figs. 2) was performed in subject specific native space, whereas the structural connectivity of the ischemic lesion pictured all fibers passing through the lesion mask. For the deterministic analysis (Figs. 3), fiber tracts were reconstructed and quantified with standard tools [13]. Because the MRI images were not strictly symmetric, a whole right thalamic mask containing the stroke lesion was secondarily made, based on the Neuromorphometric atlas [14], and its symmetric counterpart built on the left side. These anatomical masks were projected in the patient native space, and the same tracts and target areas were pictured on both sides. Interestingly, the tracts of interest in the right thalamus appeared to cross or contact the VPL (left panels of Fig. 3a and b), suggesting that this approach did not significantly distort the original stroke lesion localization and tract analysis. For quantitative data interpretation, we made the assumption that differences observed in fiber tract number between the left and the right thalamus resulted predominantly from the stroke lesion.

The probabilistic tractography (Fig. 2a and b) showed that the thalamic lesion was most strongly connected to the right occipital lobe, the parietal cortex, the superior parietal lobule, but also to the right motor cortex (including the primary motor cortex, M1). In addition, a significant deeper and more internal connectivity was seen with some parts of the right precuneus (inset in Fig. 2). Compared to the contralateral side, deterministic tractography showed a profound reduction of right thalamic fiber tracts targeting the precuneus (~ 80% reduction, Figs. 3a), the ipsilateral superior parietal lobule (SPL, ~ 90% reduction, Figs. 3b) and, to some extent, in fibers pointing to the primary somatosensory cortex (S1, ~ 60% reduction, not shown). These findings coincided with the structural connectivity pictured by probabilistic data, further suggesting that these areas were involved in stroke-related modifications and thus presumably correlated to chronic clinical findings. Connectivity to the motor cortex corresponded to the least fiber tract reduction according to the deterministic analysis (45%, data not shown). The VPL did not appear to have connection with the right posterior insula and inferior parietal lobule, whereas the superior longitudinal fasciculus (SLF) and the arcuate fasciculus were not affected at all by the thalamic lesion (data not shown).

### Clinical evolution and outcome (Fig. 1, bottom panel)

The patient benefited from multidisciplinary neurorehabilitation including physiotherapy, occupational therapy and cognitive therapy. Mild left upper limb hemiparesis and cerebellar ataxia rapidly disappeared within 1–2 weeks. Although the patient initially believed his MSG was real, he became critical about it ~ 1–2 weeks upon admission (4–5 weeks post-stroke). The patient was discharged 6 weeks post-stroke and continued with ambulatory physiotherapy and appropriate symptomatic medication (pregabalin 100 mg twice a day) in addition to secondary stroke prevention measures (see above).

Neuropsychological dysfunctions (including left hemianopia) further improved up normalization around 9 weeks post-stroke. Spontaneous MSG persisted up to 6 months post-stroke, before being evoked exclusively when the patient underwent stressful situations (anger, fear, anxiety, or when confined in a restricted space). Sensory disturbances decreased progressively over time too, consisting of mild paresthesia and pain in the left forearm at the end of the follow up (15 months post-stroke).

Overall, the patient was satisfied with the stationary and the post-discharge evolution of his symptoms and with his quality of life. He could partially (40%) go back to his formal job as a case manager and advisor in a health insurance company, despite (slowly improving) fatigue. Otherwise, he experienced blunted emotions resulting in conflictual relationship with his partner and his daughter. However, his familial situation was progressively stabilizing at the end of our follow-up, although he separated in the meantime from his partner.

## Discussion

### General considerations

We report here a 15 month-follow up of a 45-year old man known for several cardiovascular risk factors, who presented several neurological symptoms (including MSG) in the context of multiple acute ischemic stroke lesions of undetermined origin, sequentially treated with IV thrombolysis and arterial thrombectomy with stenting, out of the recommended time windows, most probably owing to his particular clinical context (young age, no severe comorbidity, sequential aggravation with persistent penumbra, arterial occlusions). In general, somatic and cognitive neurological deficits were compatible with stroke lesion localizations. Motor, cerebellar, visual field and cognitive deficits rapidly normalized concomitantly with disappearance of lesions on brain imaging, while left-sided MSG and sensory disturbances persisted over months in parallel with a residual right thalamic lesion. Progressive functional improvement in stationary Neurorehabilitation led to discharge back home and to partial return to work.

### Stroke etiology of MSG

Several pathologies can lead to MSG [4, 15]. Our patient did not suffer from migraine, infection or any active psychiatric disease at the time of stroke. The clinical constellation did not suggest an ictal origin neither, although no electroencephalogram was performed. Thus, the only plausible etiology of MSG appeared to be stroke lesion. In support to this assumption, MSG persisted in parallel with left-sided sensory symptoms that were anatomically compatible with the residual right thalamic ischemic lesion. To our best knowledge, there is no previous report of MSG due to thalamic lesions, BSD being traditionally associated to (right) parietal lesions [10] or more widely to lesions of the temporoparietal-occipital carrefour [4]. Of notice, focal lesions constitute a rare cause of MSG (< 10%), with stroke representing less than one third of the cases [3].

### Anatomical substrate of MSG

While the anatomo-clinical coherence between the main neurological symptoms (left homonymous hemianopia, ataxia and left-sided sensory symptoms) and identified lesions (right occipital, cerebellar and right thalamic lesions) was clear, lesion in the (right) parietal cortex, most generally reported in MSG [10], lacked in our patient. Furthermore, the other incriminated brain areas (the frontal lobe, the posterior insula, the superior and inferior parietal lobules, the precuneus) [9–11], owing to their connections with the parietal cortex, were not affected neither.

Among the patient’s lesions, tractography data showed that only the thalamic lesion shared anatomical link with the parietal cortex. Yet, within the thalamus, the VPL (where the lesion was most probably located) constitutes an important provider of thalamic connections targeting the superior parietal lobule and the precuneus [8, 9, 12] (both involved MSG). Interestingly, our data not only confirmed this strong connectivity, but they showed, in addition, a drastic reduction in fiber tracts number between the VPL ischemic lesion and both areas in comparison to the left side. The SLF, which supposedly underlies the association between the frontal lobe and MSG [9, 10], was not damaged in our patient. Likewise, no connection was seen with the posterior insula and the inferior parietal lobule, most probably because they do not receive projections from the VPL [16]. The strong connectivity observed with the motor cortex could either be related to altered functional connection between the VPL and M1 [12] or to a wallerian degeneration explaining the modest reduction in fiber tracts targeting the M1 area. Overall, M1 involvement appeared to have minor clinical significance, apart from the initial transient mild left hemiparesis and MSG enhancement induced by the affected limb movements.

In summary, brain imaging data disclose the right VPL as an essential relay mediating appearance of MSG, even in absence of direct lesion of the parietal lobe, thereby suggesting a “diaschisis”-like mechanism engaged.

### Mechanisms underlying MSG

Tractography showed significant structural connectivity coinciding with important reduction of fiber tract number between the thalamic lesion and S1. The prominent left-sided sensory dysfunction further confirmed the role of VPL as a major thalamic relay of somatosensory inputs unto S1 [17]. Whether sensory deficits contributed to MSG constitutes an interesting question, especially if we consider that both symptoms improved in parallel and affected the same body parts.

Peripheral sensory deafferentation/lesion, one mechanism that could mediate this association [15, 18], was not observed in our patient. Alternatively, MSG could be a result of disordered primary or high order neural processing of body representation [15, 18] taking place in the VPL, as suggested by experimental animal results [19]. Defective integration of visual and somatosensory inputs [3] could also be discussed. In previous reports, patients who criticized their MSG [10] or witnessed MSG disappearance under visual control [9] had intact SPL (a structure where integration of visual and somatosensory information takes place), contrary to the patient reported here in whom a significant reduction of fibers targeting the SPL was observed. The role played by the observed mild executive dysfunction in the lack of criticism towards MSG could also be questioned. However,not only it outlasted the moment when the patient became critical regarding his MSG, but it had no other behavioral consequence.

### Classification of MSG

According to his description of MSG, the patient had hyperschematia (enlarged body parts size; included among aschematia) and paraschematia (displacement of body parts position; noticed when his eyes were closed) [5], thereby fulfilling criteria for the rare (< 10%) type A of AIWS [4]. However, if we take into account the delusional-like feature (not typical for MSG [20]), he could be as well considered as type C (MSG + delusion) although delusions are typically much more florid and prominent in this group [4]. Thus, the phenomenology of MSG in this patient seems to represent an intermediate type of AIWS not individualized in existing classifications. The later tend to separate (sometimes even oppose) somesthetic (cognitive) and psychic (delusional) symptoms [3, 4], while the wide variety of semiological descriptions makes this distinction challenging for an inclusive classification. Perhaps AIWS should be considered as a continuous spectrum including on one extreme, predominantly cognitive (non-delusional) forms due to structural brain (focal) lesions, and on the other extreme, more delusional (psychic) profiles without individualized lesion on brain imaging. This new perspective would allow integration of intermediate profiles and more comprehensive pathophysiological considerations.

## Conclusion

We report the case of a patient presenting mainly with long-lasting left-sided MSG and sensory deficits presumably associated with a right thalamic stroke lesion restricted to the VPL, that progressively improved over months. Tractographic data pointed out the essential role of thalamic connections to the parietal cortex (especially the precuneus) via the VPL, a crucial nucleus in body size coding and perception. Damaged SPL projections of the VPL might have contributed to the lack of criticism towards MSG and absence of visual control of MSG. Defective neural processing and integration of sensory information by and/or through the VPL seems to be essential in generation of MSG. The patient’s MSG was classified as an intermediate (non-individualized) stage between the types A and C AIWS. Further discussion on MSG phenomenology is needed, considering AIWS as a continuum flexibly associating cognitive and psychic symptoms, and thereby possibly featuring an alternative classification.

The case reported here was well-documented during and after the neurorehabilitation stay, which allowed consistent anatomo-clinical discussion. However, detailed description of the patient’s initial neurological complains, including a precise time when the main symptoms discussed in the paper started, lacked. In addition, tractography data were obtained in the chronic phase of long-lasting neurological symptoms and lesions, making it difficult to extrapolate with certainty correlation between acute ischemic lesions and initial symptoms. Thus, our findings have to be considered with caution, especially when generalizing derived conclusions. Nevertheless, this first detailed analysis of MSG in the context of a non-cortical brain lesion explores interesting hypotheses that would highly contribute, if confirmed, to understand mechanisms underlying this unusual symptom, as well as the role of the VPL.

## Supplementary information

**Additional file 1.**

**Additional file 2.**

## Data Availability

All data and materials related to this study are ready to be made available from the Neurology Unit server in a format that does not allow recognition of the reported patient.

## Abbreviations

AIWS: Alice in wonderland syndrome
BSD: Body Schema Disorders
CPAP: Continuous Positive Airway Pressure
CT: Computerized Tomography
DTI: Diffusion Tensor Imaging
DWI: Diffusion Weighted Imaging
ED: Emergency Department
LDL: Low Density Lipoproteins
M1: Primary motor cortex
MRI: Magnetic Resonance Imaging
MSG: Macrosomatognosia
MoCA: Montreal Cognitive Assessment
NIHSS: National Health Institute Stroke Scale
PCA: Posterior Cerebral Artery
S1: Primary somatosensory cortex
SLF: Superior Longitudinal Fasciculus
SPL: Superior Parietal Lobule
VPL: Ventral posterolateral nucleus
