# Supplementary material for: The right thalamic ventral posterolateral nucleus seems to be determinant for macrosomatognosia: a case report
Source: BMC Neurol. 2020 Oct 28;20:393. doi: 10.1186/s12883-020-01970-3 (PMC7594440; doi:10.1186/s12883-020-01970-3)
Supplement: Supplementary file 1 — Additional file 1. [file 12883_2020_1970_MOESM1_ESM.docx]

***Figure***


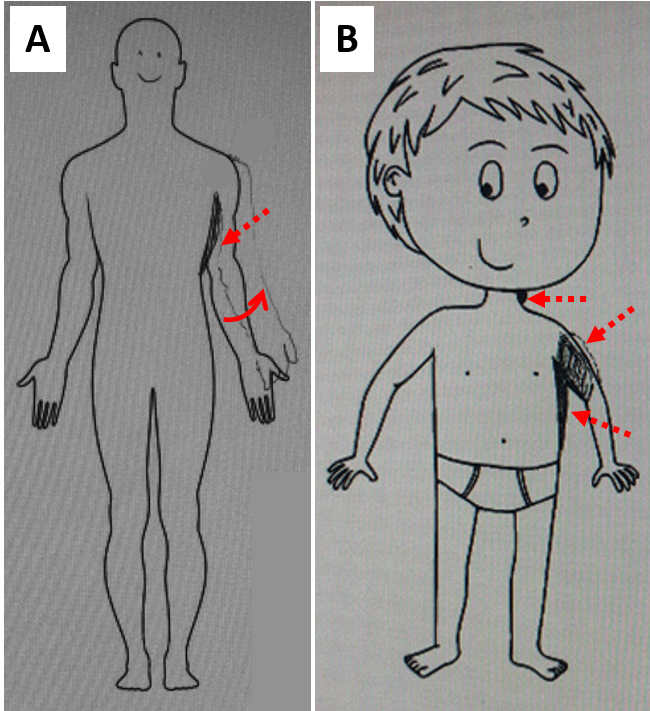


***Figure 2. Illustrative cartoons of the body parts involved in macrosomatognosia (MSG).*** (the patient made his drawings on existing templates). (**A)** On arrival in Neurorehabilitation (20 days post-stroke), the patient felt his whole upper limb as increased in size, but also as spontaneously abducted (red arrow) due to the left flank “balloon” (red discontinued arrow) (human body template adapted from <https://stock.adobe.com/fr/images/full-length-front-back-silhouette-of-a-man/43024972>, 11.2017). A ~10 cm external abduction of the left arm triggered MSG aggravation. (**B)** At 4-5 weeks post-stroke, when requested to further precise the body parts that he perceived as enlarged, he pointed respectively to the left lateral side of the neck, the left arm and the entire left lateral part of the trunk (red discontinued arrows) (body cartoon adapted from <https://www.mysticlolly.fr/dessins-le-schema-corporel/>, 11.2017). Macrosomatognosia was now restricted to the arm.
