# Supplementary material for: The right thalamic ventral posterolateral nucleus seems to be determinant for macrosomatognosia: a case report
Source: BMC Neurol. 2020 Oct 28;20:393. doi: 10.1186/s12883-020-01970-3 (PMC7594440; doi:10.1186/s12883-020-01970-3)
Supplement: Supplementary file 2 — Additional file 2. [file 12883_2020_1970_MOESM2_ESM.docx]

*Brain MRI with Diffusion Tractography detailed methods*

The last brain imaging (Figure 1C) included also a DWI sequence for tractography purposes (Figures 2 and 3). Sixty-four (64) axial slices were acquired with a matrix size of 128 x 128 (interpolated to 256x256), FOV = 26 cm, inter slice spacing = 0.2 mm, TR = 8000 ms, TE = 86.9 ms, flip angle = 90°, parallel phase acceleration factor = 2, 64 non-collinear directions with b-value=1000 s/mm2, one b=0 image. These data were corrected for eddy current and movements artefacts with validated FSL software [[1](#_ENREF_1)]. The thalamic lesion mask was drawn using MRIcro software [[2](#_ENREF_2), [3](#_ENREF_3)].

The whole-brain unconstrained probabilistic tractography (Figure 2) was performed using FSL software with a standard procedure [[4](#_ENREF_4)]. For better visualization, connectivity values were normalised to fit values between 0 to 1, and thresholded at >0.1, in order to only keep only the most significant connections.

For the deterministic analysis (Figure 3), fiber tracts were reconstructed with the Diffusion Toolkit Software (v 0.6.4.1) and quantified with the TrackVis software (v 0.6.1) [[5](#_ENREF_5)]. The anatomical masks were projected in the patient native space using backward deformation field of the T1 segmentation computed by SPM software [[6](#_ENREF_6)].

**REFERENCES**

1. Jenkinson M, Beckmann CF, Behrens TEJ, Woolrich MW, Smith SM. FSL. NeuroImage.2012;62 2:782-90; doi: https://doi.org/10.1016/j.neuroimage.2011.09.015.

2. Roden C, Brett M. Stereotaxic display of brain lesions. Behavioral Neurology. 2000;12:191-200.

3. Anonymous. https://[www.nitrc.org/projects/mricron/](http://www.nitrc.org/projects/mricron/). Accessed 2019.

4. Behrens TEJ, Berg H, Jbabdi S, Rushworth MFS, Woolrich MW. Probabilistic diffusion tractography with multiple fibre orientations: What can we gain? NeuroImage. 2007;34:144-55; doi: 10.1016/j.neuroimage.2006.09.018.

5. Wang R, Wedeen V. <http://trackvis.org/> (2019). Accessed 2019.

6. Anonymous. <http://www.fil.ion.ucl.ac.uk/spm>. Accessed 2019.
